# Migration of Ethylene Vinyl Alcohol Co-Polymer in the Urinary Tract Successfully Managed

**DOI:** 10.3390/medicina55060234

**Published:** 2019-05-31

**Authors:** Anna Maria Ierardi, Filippo Pesapane, Antonio Arrichiello, Federico Fontana, Filippo Piacentino, Gianpaolo Carrafiello

**Affiliations:** 1 Diagnostic and Interventional Radiology Department, University of Milan, Via di Rudinì 8, 20142 Milan, Italy; amierardi@yahoo.it (A.M.I.); arrichielloantonio@gmail.com (A.A.); 2 Postgraduate School in Radiodiagnostics, Università degli Studi di Milano, Via Festa del Perdono 7, 20122 Milan, Italy; 3 Radiology Department, Insubria University, Viale Borri 57, 21100 Varese, Italy; fede.fontana@libero.it (F.F.); f.piacentino@live.it (F.P.); 4 Radiology Department, Foundation IRCCS Cà Granda-Ospedale Maggiore Policlinico, Via Francesco Sforza 28, 20122 Milan, Italy; gcarraf@gmail.com

**Keywords:** interventional radiology, EVOH, onyx, pseudoaneurysm, migration, complication

## Abstract

Selective embolization is the treatment of choice for traumatic renal pseudoaneurysm. The use of ethylene vinyl alcohol copolymer (EVOH) was recently described as an embolic agent in peripheral lesions. The aim of a good embolic agent is to: achieve rapid and effective embolization; reach and fill distal vasculature targeted for embolization; be easy to prepare and use. Moreover, it should be highly radiopaque, controllable during administration, biocompatible and cost-effective. EVOH is a non-adhesive embolic agent and its efficacy is independent from the coagulant status. The risk of non-targeted embolization should be reduced by the good radio-opacity of the embolic material that is injected under continuous fluoroscopy. Nevertheless, symptomatic EVOH migration was described. We report a unique case of embolization of a renal pseudoaneurysm and migration of EVOH in the urinary tract. Retrograde trans-urethral removal of the migrated embolic agent was successfully performed. Our case report indicates that EVOH may not be appropriate when a fistula with renal calyx is suspected, even if its migration in the urinary tract may be managed.

## 1. Introduction

Renal vascular lesions may be caused by blunt or penetrating trauma. Arteriovenous fistula and pseudoaneurysm are the most common renal vascular injuries after blunt trauma. Further causes may be iatrogenic injuries during a procedure or spontaneous lesions with or without underlying pathology [1]. The decision whether or not to repair this type of lesion depends on the blood flow through the fistula, the patient’s clinical conditions (e.g., the presence of heart failure or arterial hypertension), the risk of rupture in the case of large false aneurysms, and recurrent hematuria. Particularly, in case of massive hemorrhage or continuous hematuria, aggressive therapy is needed [2]. Since the first trans-arterial renal embolization in 1964, the devices used in interventional radiology have evolved: super selective embolization is now possible with minimal tissue loss, and it is considered to be the first line of treatment [3]. The choice of the embolic agent is not stabilized by specific guidelines, and the final decision is based on the operator’s experience and confidence [4].

The aim of a good embolic agent is to: achieve rapid and effective embolization; reach and fill distal vasculature targeted for embolization; be easy to prepare and use. Moreover, it should be highly radiopaque, controllable during administration, biocompatible and cost-effective [5].

The most common coils are made of steel or platinum, since they are a permanent embolic agent; their fibers induce clot formation. For selective arterial embolization, it is possible to also use different agents, such as polyvinyl alcohol (PVA) particles, acrylic polymers, microspheres, alcohol, and n-butylcyanoacrylate (NBCA) [4,6].

Onyx (Onyx Liquid Embolic System; ev3 Neurovascular, Irvine, CA, USA) is a non-adhesive material, unlike other liquid embolic agents. Onyx is a biocompatible polymer (ethylene vinyl alcohol copolymer (EVOH)) that is dissolved in an organic solvent, dimethyl-sulfoxide (DMSO) [7]. It features less adhesiveness and slow polymerization: consequently, EVOH is able to fill the spaces (endoleaks, aneurysm, pseudoaneurysm, arterial branch feeding the bleeding, nidus of malformations, etc.) [4,8,9,10,11,12,13].

Onyx was successfully used for the embolization of renal arteriovenous malformations, renal aneurysms and pseudoaneurysms [14,15].

The risk of non-targeted embolization should be reduced by the good radio-opacity of Onyx and its injection under continuous fluoroscopy. Nevertheless, symptomatic EVOH migration in pulmonary arteries was described after embolization of cerebral artero-venous malformations (AVM), in the graft limb during embolization of Type I endoleak (EL) and during gastrointestinal (GI) bleeding causing non-target embolization [13,16,17].

We describe an unusual case of embolization of a renal pseudoaneurysm (PSA) and migration of EVOH in the urinary tract and a consequently successful retrograde recovery through the bladder.

## 2. Case Presentation

A 67-year-old male with 15 days macrohematuria and consequent anemization was presented. Nothing of relevant was collected from his clinical history, despite the use of chronic aspirin as secondary prevention due to previous myocardial ischemic events that did not require interventions. Regarding the anamnesis, he reported a recent (about 20 days earlier) but non-relevant trauma falling from the stairs.

Our Internal Review Board approved the retrospective analysis of this interventional procedure (OSP2015/2495). Any further consent was not expected: EVOH is an embolic agent approved and usually used for peripheral embolization.

**Figure 1:**
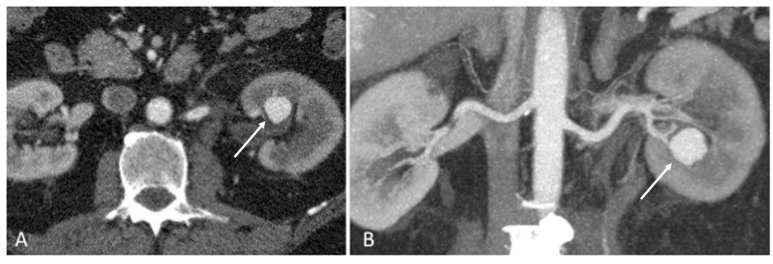
A multi phases CT revealed a left pseudoaneurysm (arrow) (**A**); Coronal Maximum Intensity Projection (MIP) reconstruction confirmed the finding (arrow) (**B**).

The urologist proposed to our Radiology Department to proceed with an ultrasound that was not diagnostic. Then, a CT scan was proposed. A multi phases CT revealed a left pseudoaneurysm (Figure 1).

**Figure 2:**
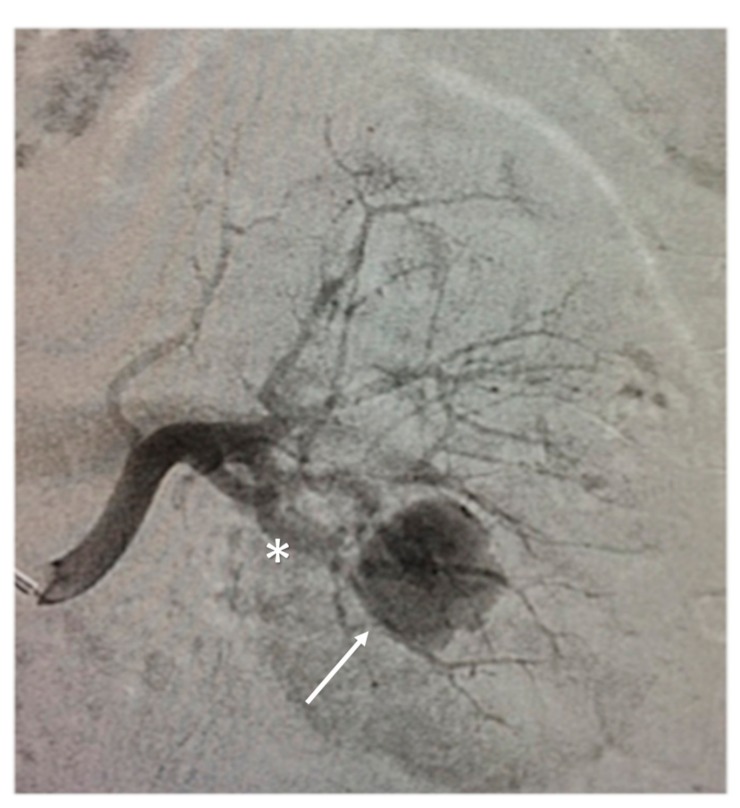
Angiogram confirmed pseudoaneurysm (arrow) with its afferent vessel (asterisk).

The patient was proposed to our Interventional Radiology (IR) Unit for transarterial embolization. First angiogram confirmed pseudoaneurysm; selective catheterization of the feeder was obtained (Figure 2). Due to the optimal position of the microcatheter, the operator chose EVOH Onyx 34 (Medtronic plc, Dublin, Ireland) as an embolic agent. The volume of EVOH injected was about 0.3 mL, with an injection rate of about 0.15 mL/min. Additionally, 0.7 mL of dimethyl-sulfoxide DMSO was used to fill the dead space of the microcatheter. No adverse reactions were reported in the patient. In our institution, we are currently performing a prospective collection of patients with altered coagulant status and renal traumatic or iatrogenic pseudoaneurysms treated with EVOH (usually Onyx 34), in order to compare the safety and effectiveness of them with the cases treated with other embolic agents.

**Figure 3:**
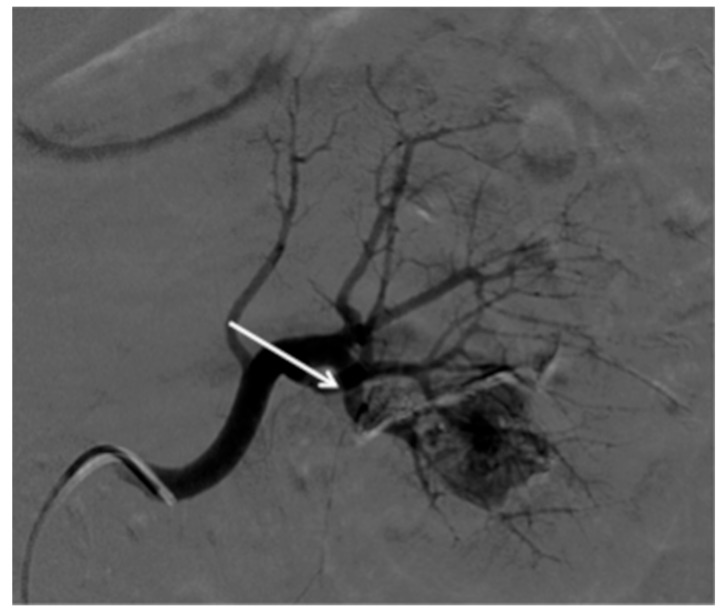
During the procedure of embolization, an extravasation of the ethylene vinyl alcohol copolymer was observed, and a slow filling of the inferior renal calyx was observed (arrow).

During the injection of EVOH, an extravasation of the embolic agent was observed, and a slow filling of the inferior renal calyx was observed (Figure 3). Migration and incomplete embolization of the PSA have been confirmed by the angiogram. The procedure was concluded through microcoils. The patient was stable and asymptomatic during the entire procedure.

**Figure 4:**
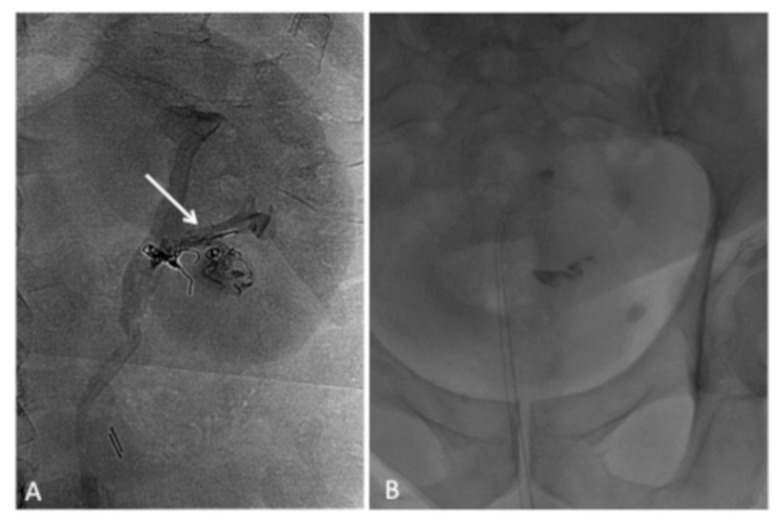
Single shot acquisition demonstrating the presence of microcoils, EVOH in part in the PSA and remnant in the inferior renal calyx (arrow) (**A**); single shot of the pelvis revealed the presence of EVOH in the projection of the bladder (**B**).

**Figure 5:**
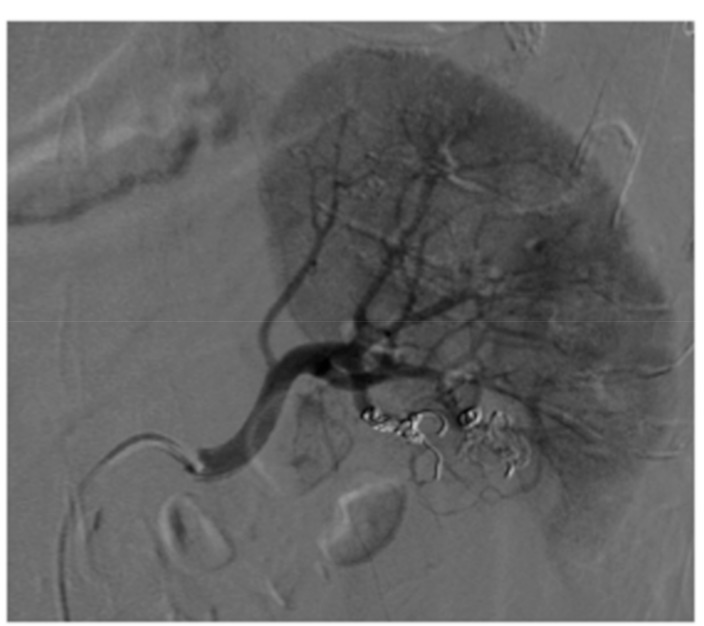
Renal angiogram confirmed complete exclusion of the PSA.

Given the stability of these parameters and the disappearance of the hematuria, the patient was referred to our IR unit to remove the sheath after 2 days. Due to accidental migration of EVOH during procedure, a single shot on left flank was acquired. EVOH was no longer observed in the renal calyx, while it was shown in the bladder by a further single shot of the pelvis (Figure 4). Complete exclusion of the PSA has been confirmed through a renal angiogram (Figure 5).

**Figure 6:**
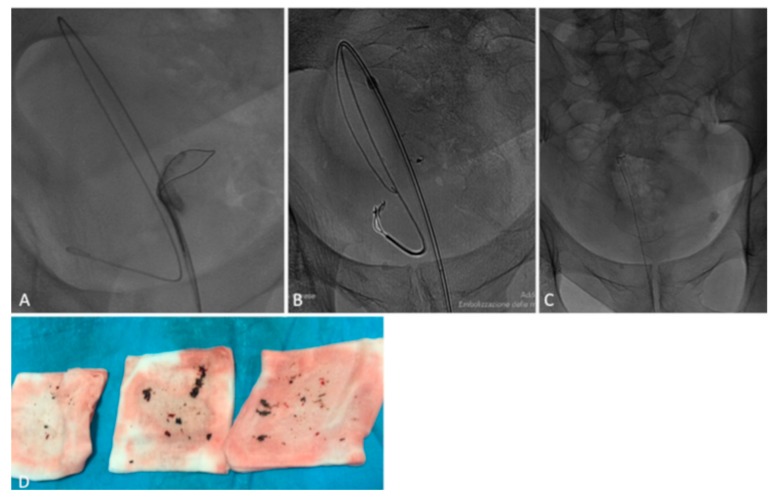
A retrograde removal of EVOH was performed using a goose neck catheter (**A**,**B**); the embolic agent was successfully completely removed at the final image (**C**); image shows EVOH removed (**D**).

Although the patient was completely asymptomatic, the operator, according to the patient, decided to approach the bladder per via retrograde to attempt the removal of EVOH migrated. The embolic agent was then completely removed using a goose neck catheter (Figure 6).

**Figure 7:**
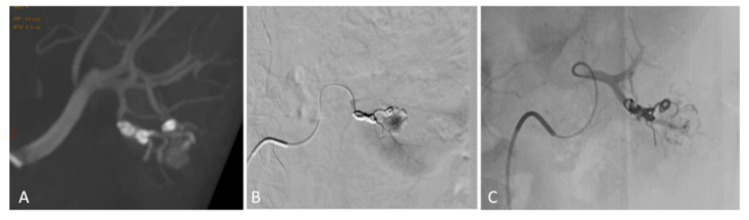
Intra-procedural CBCT (**A**) and arteriogram (**B**) respectively confirmed blood penetration through the coils. One millimeter of NCBA (1:1) was selectively injected to stop the flow behind the coils. Final angiogram, after embolization with one millimeter of NCBA (1:1), showed complete exclusion of PSA (**C**).

Two days later, the patient was discharged as urine was clear and he was asymptomatic. However, one week later, patient presented hematuria again; he was then admitted to our Urology Department to perform a new CT. Pseudoaneurysm was in part refilled. A new arteriography and intra-procedural cone beam CT (CBCT) were performed: the results confirmed blood penetration through the coils. To stop the flow behind the coils, one millimeter of N-butyl-2-cyanoacrylate (NBCA) (1:1) has been selectively injected. Final angiogram showed complete exclusion of PSA (Figure 7). After 3 days, the patient has been discharged; hematuria no longer appeared in the available follow up of 12 months.

## 3. Discussion

Different factors may affect the efficacy of embolization: the patient’s clot-forming ability, the site of the bleeding and the local vasospasm. The embolization efficacy of EVOH is independent of a normal coagulation status, and the rate of clinical failure after embolization is higher when the patient has coagulopathy [4,13].

In similar situations, compared to other embolic agents, this represents an advantage.

Our institution’s experts perform a great number of emergency embolization every year. Two years ago, we collected information from consecutives patients with iatrogenic renal vascular injuries treated using transcatheter embolization [2]. Embolization was obtained with micro coils, embospheres, polyvinyl alcohol particles, spongostan emulsion and vascular plug.

Most injuries due to blunt trauma consist of lower-grade, non-life-threatening injuries [1]. Renal injuries may be divided into renal laceration, renal contusion, and renal vascular injury. Arteriovenous fistula and pseudoaneurysm are the most common renal vascular injuries after blunt trauma and the risk of aneurysms’ rupture and the recurrent hematuria may request aggressive treatment. Over the last year we started performing a prospective collection of patients with renal iatrogenic or traumatic pseudoaneurysms treated with EVOH: we are going to compare retrospectively safety and effectiveness of different embolic agents; due to chronic antiplatelet or anticoagulant therapies, all the patients included are affected by alteration of their coagulation status.

Moreover, onyx is the embolic agent of choice to overcome the problems of endovascular navigation and to first obtain a distal occlusion, and then a proximal occlusion [4,18,19,20].

Considering its ability to penetrate, it is essential to pay extremely attention to the progression of the EVOH when potentially dangerous anastomoses and/or fistulas are present [21].

According to previous studies, EVOH was used to embolize arteriovenous malformations, renal aneurysm and pseudoaneurysms, angiomyolipoma and arteriovenous fistulas [4,19,22,23].

During elective or urgent renal embolization, urinary tract migration of onyx had never been described before.

In literature, migration of onyx in other vascular districts was described: in pulmonary arteries after embolization of cerebral AVM, in the graft limb during embolization of Type I EL and during GI bleeding causing bowel ischemia [13,16,17].

In these cases, maybe because of unrecognized connections or strange hemodynamic flows or technical imperfections, there was a migration of onyx through the vascular district. In the case presented, the only symptom of the patient was macrohematuria. An arterio-calyceal fistula was present. On the basis of literature data, another probable arterio-calyceal fistula have been successfully treated with EVOH [22]. The case described in this study, represents the first instance of migration in the urinary tract; the patient was asymptomatic, he did not even refer colic pain or any other symptoms. Operators preferred to remove the embolic agent via a retrograde approach of the bladder because of the risk of unknown consequences.

The refilling of the renal PSA by blood penetration through the coils previously deployed may be related to two reasons: the coagulant status of the patient and the migration of the embolic agent.

## 4. Conclusions

EVOH may not be appropriate when a fistula with renal calyx is suspected, even if its migration in the urinary tract may be managed.
